# A decade of iron overload disorders and hemochromatosis: clinical and genetic findings from a specialized center in Colombia

**DOI:** 10.3389/fmed.2024.1494527

**Published:** 2024-12-10

**Authors:** L. C. Quiroga Cristancho, María Alejandra Urbano, H. A. Nati-Castillo, María Alejandra Obando, Rigoberto Gómez-Gutiérrez, Juan S. Izquierdo-Condoy

**Affiliations:** ^1^Department of Internal Medicine, Interinstitutional Internal Medicine Group (GIMI 1), Universidad Libre, Cali, Colombia; ^2^Departamento de Hematología, Hemato Oncólogos S.A, Cali, Colombia; ^3^One Health Research Group, Univerisdad de las Americas, Quito, Ecuador

**Keywords:** iron overload disorders, hemochromatosis, clinical characterization, mutations, low-and middle-income countries, South America

## Abstract

**Background:**

Iron overload disorders, including hereditary hemochromatosis (HH), are characterized by excessive iron accumulation, which can cause severe organ damage. HH is most associated with the C282Y mutation in Caucasian populations, but its prevalence and genetic profiles in Latin American populations remain underexplored.

**Objectives:**

To describe the clinical manifestations, genetic profiles, and biochemical characteristics of patients with suspected iron overload disorders in a specialized hematology center in Cali, Colombia.

**Methods:**

A retrospective observational study was conducted on 70 patients diagnosed with iron overload disorders between 2014 and 2024. Data on clinical presentation, laboratory results, imaging, and genetic mutations were collected. Statistical analyses, including chi-square tests and logistic regression, were used to evaluate factors associated with HH diagnosis.

**Results:**

Male patients constituted 64.3% of the sample, with a mean age of 56.1 years at diagnosis. Fatigue (27.1%) and joint pain (17.1%) were the most common symptoms. Of the total sample, 32.9% were diagnosed with hemochromatosis. The H63D mutation was the most prevalent (52.2%), while the C282Y mutation was rare. A predominance of both slight (100.0%) and limitrophe (58.3%) iron overload was identified among patients with hemochromatosis (*p* = 0.036).

**Conclusion:**

Colombian patients with iron overload disorders show clinical, epidemiological, and biochemical profiles consistent with global patterns, yet exhibit distinct genetic diversity. Notably, they have a low prevalence of the C282Y mutation and a higher prevalence of the H63D mutation, differing from European HH profiles. Despite elevated ferritin and transferrin saturation, no significant clinical symptoms were observed, suggesting potential delays in diagnosis. These findings highlight the need for early, region-specific diagnostic approaches to prevent complications like cirrhosis and underscore the importance of further genetic research across Latin America.

## 1 Introduction

Iron overload disorders, both acquired and inherited, represent a significant challenge in clinical practice due to their potential to cause widespread organ damage (1). Acquired causes of iron overload include chronic viral hepatitis, thalassemia, and alcoholic liver disease, which are more common than hereditary hemochromatosis (HH) (2). However, HH remains the best-described inherited disorder of iron metabolism. This genetic condition is mainly characterized by aberrant regulation of iron absorption, especially mediated by hepcidin inhibition, leading to increased systemic iron levels and causing serious damage to end organs, such as arthropathy, skin hyperpigmentation, diabetes, liver fibrosis, cirrhosis, and hepatocellular carcinoma (3, 4). The majority of Caucasian patients with HH present homozygosity for the C282Y mutation in the HFE gene, although a significant proportion remain clinically silent despite abnormal iron indices (5).

Excess iron deposition in organs such as the liver, heart, and endocrine glands can generate reactive oxygen species, causing cellular damage and associated symptoms (6). An estimated 16 million Americans experience some degree of iron overload, and hereditary hemochromatosis predominantly affects people of European ancestry (7, 8). Relevant studies in various populations, such as those in the United States and Canada, have shown variable prevalence rates of the C282Y mutation (9). However, the lowest prevalences have been identified in Black and Mexican American individuals (between 2.3 and 2.8%) (7, 10), highlighting the genetic diversity and geographical differences in the occurrence of HH.

Despite the global recognition of HH, the characterization of iron overload disorders in specific populations, such as those in Latin America, remains limited. In Colombia, particularly, the clinical, epidemiological, and genetic behavior of iron overload disorders is mainly documented in case reports (11, 12). Therefore, this study aims to describe the clinical manifestations, genetic profiles, and paraclinical characteristics of patients with suspected iron overload disorders in a specialized hematology center in Cali, Colombia, during the last decade.

## 2 Materials and methods

### 2.1 Study design

A retrospective observational study was conducted based on the analysis of clinical records from Hemato Oncólogos S.A., a specialized care center for hematological diseases, serving the departments of Valle del Cauca and Cauca. This center is considered a reference for the southwestern territory of Colombia.

### 2.2 Population

The southwestern region of Colombia is composed of four departments: Valle del Cauca, Cauca, Nariño, and Putumayo. The first two contain 75% of the population, with approximately 6,150,208 inhabitants, according to projections by the National Administrative Department of Statistics (DANE) for 2023 (13). Hemato Oncólogos S.A. is a specialized center that serves many people from both the public and private sectors of the Health Promotion Entity (EPS) from Valle del Cauca and Cauca, two of the most important departments in southwestern Colombia.

For this study, the medical records of patients treated in the hematology service under the suspected diagnosis of iron metabolism disorder between January 2014 and July 2024 were reviewed.

### 2.3 Sample

Data collection was carried out through non-probabilistic sampling using consecutive medical records. The sample included all patients seen in the hematology service of Hemato Oncólogos S.A. during the specified study period who met the selection criteria.

### 2.4 Inclusion and exclusion criteria

The inclusion criteria were based on the inspection of medical records of patients over 18 years of age referred to the hematology clinic with ICD-10 diagnoses (E83.1: Disorders of iron metabolism, E83.19: Other disorders of iron metabolism, and E83.119: Hemochromatosis, unspecified) between January 2014 and July 2024, who had medical follow-up for at least 1 year at the institution. Patients under 18 years of age, records falling outside the study period, those with insufficient data due to poor quality of clinical reports, and those who were already in follow-up with a confirmed diagnosis of hemochromatosis were excluded.

### 2.5 Sample and data collection

After receiving authorization, 77 medical records with the previously described ICD-10 diagnoses were accessed from the anonymous source database of Hemato Oncólogos S.A. during the specified period. After applying the inclusion and exclusion criteria, 7 records were eliminated (2 due to lack of information in the medical history, 1 due to follow-up of less than 1 year, 2 due to follow-up time prior to 2014, and 2 due to a previously confirmed diagnosis of hemochromatosis), resulting in a final sample of 70 valid medical records.

From the included records, an anonymous database was constructed that included data on demographic variables such as sex, age at follow-up, age at diagnosis, initial ferritin values, and blood transferrin saturation. It also included the last follow-up ferritin value and transferrin saturation, clinical variables such as history of type 2 diabetes mellitus, symptoms related to hemochromatosis (arthralgia, fatigue), abdominal ultrasound analysis, magnetic resonance imaging (MRI) analysis of the abdomen, genetic mutation, and treatment.

### 2.6 Statistical analysis

The different variables were described using frequencies and percentages. For numerical variables, the distribution of the data was evaluated using the Shapiro–Wilk test. Measures of central tendency, including mean and standard deviation, were subsequently used for description. Chi-square tests were used to examine the relationship between qualitative variables, and Student’s t-test was used to identify differences in means between numerical variables and the diagnosis or non-diagnosis of hemochromatosis. Finally, a logistic regression model was used to identify factors associated with the diagnosis of hemochromatosis. A *p*-value <0.05 was considered statistically significant. All data analyses were performed using IBM SPSS Statistics for Windows, version 29.0 (IBM Company, Chicago, IL, United States).

### 2.7 Ethical statement

All research procedures were in accordance with local regulations and adhered to the principles described in the Declaration of Helsinki. In addition, the study received approval from the Centro de Investigación en Cancer de Hemato Oncólogos S.A. – CIHO.

## 3 Results

### 3.1 Demographic and clinical characteristics of iron overload

A total of 70 patients were included in this study. The majority were male, comprising 64.3% (*n* = 45), with a mean age at diagnosis of 56.1 ± 12.8 years and a mean follow-up of 4.3 ± 3.1 years. Among the patients, a history of diabetes mellitus and liver cirrhosis was identified in only one patient (1.4%). Clinically, 27.1% of patients (*n* = 19) presented with fatigue, 17.1% (*n* = 12) with joint pain, and the remaining patients were asymptomatic (Table 1).

**Table 1:** Demographic and clinical characteristics of patients with iron overload over 10 years.

	Mean	± SD
Age at diagnosis (years)	56.1	12.8
Current age (years)	60.4	12.5
Follow-up time (years)	4.3	3.1

	*n*	%
Sex		
Female	25	35.7%
Male	45	64.3%
Diabetes mellitus 2		
No	69	98.6%
Yes	1	1.4%
Liver cirrhosis		
No	69	98.6%
Yes	1	1.4%
Joint pain		
No	58	82.9%
Yes	12	17.1%
Fatigue		
No	51	72.9%
Yes	19	27.1%

The analyses of the participants showed a mean ferritin value at first contact of 888.2 ± 1087.8 μg/L, which decreased to a mean ferritin value at last contact of 524.5 ± 399.7 μg/L. A similar trend was observed in transferrin saturation, which went from 70.8 ± 208.0% initially to 39.6 ± 14.1% in the final measurement (Table 2). Imaging scans showed a predominance of mild hepatic steatosis in 34.3% (*n* = 24), while liver MRI analysis revealed borderline liver iron content in 17.1% (*n* = 12) and normal hepatic iron content in 25.7% (*n* = 18; Table 2). Of the total patients included, 23 (32.9%) were diagnosed with hemochromatosis. Among the population analyzed, the preferred treatment was based on the use of statins and dietary control in 21.4% (*n* = 15; Table 2).

**Table 2:** Analytical and management parameters of patients with iron overload for 10 years.

	Mean	± SD
Initial ferritin (μg/L)	888.2	1087.8
Last Ferritin (μg/L)	524.5	399.7
Initial Transferrin Saturation (mg/L)	70.8	208.0
Last Transferrin Saturation (mg/L)	39.6	14.1

	*n*	%
Hepatic ultrasound		
Normal	10	14.3%
Mild hepatic stheatosis	24	34.3%
Moderate hepatic stheatosis	9	12.9%
Severe hepatic stheatosis	1	1.4%
N/A	26	37.1%
Liver iron content		
Normal	18	25.7%
Slight overload	1	1.4%
Limitrophe overload	12	17.1%
N/A	39	55.7%
Hemochromatosis diagnosis		
No	47	67.1%
Yes	23	32.9%
Treatment		
Deferasirox	3	4.3%
Diet	12	17.1%
Diet/Statin	15	21.4%
Statin	2	2.9%
Phlebotomy	7	10.0%
Phlebotomy/Diet	8	11.4%
Phlebotomy/Statin	4	5.7%
Phlebotomy/Statin/Diet	6	8.6%
None	13	18.6%

### 3.2 Factors associated with the diagnosis of hemochromatosis

**Figure 1:**
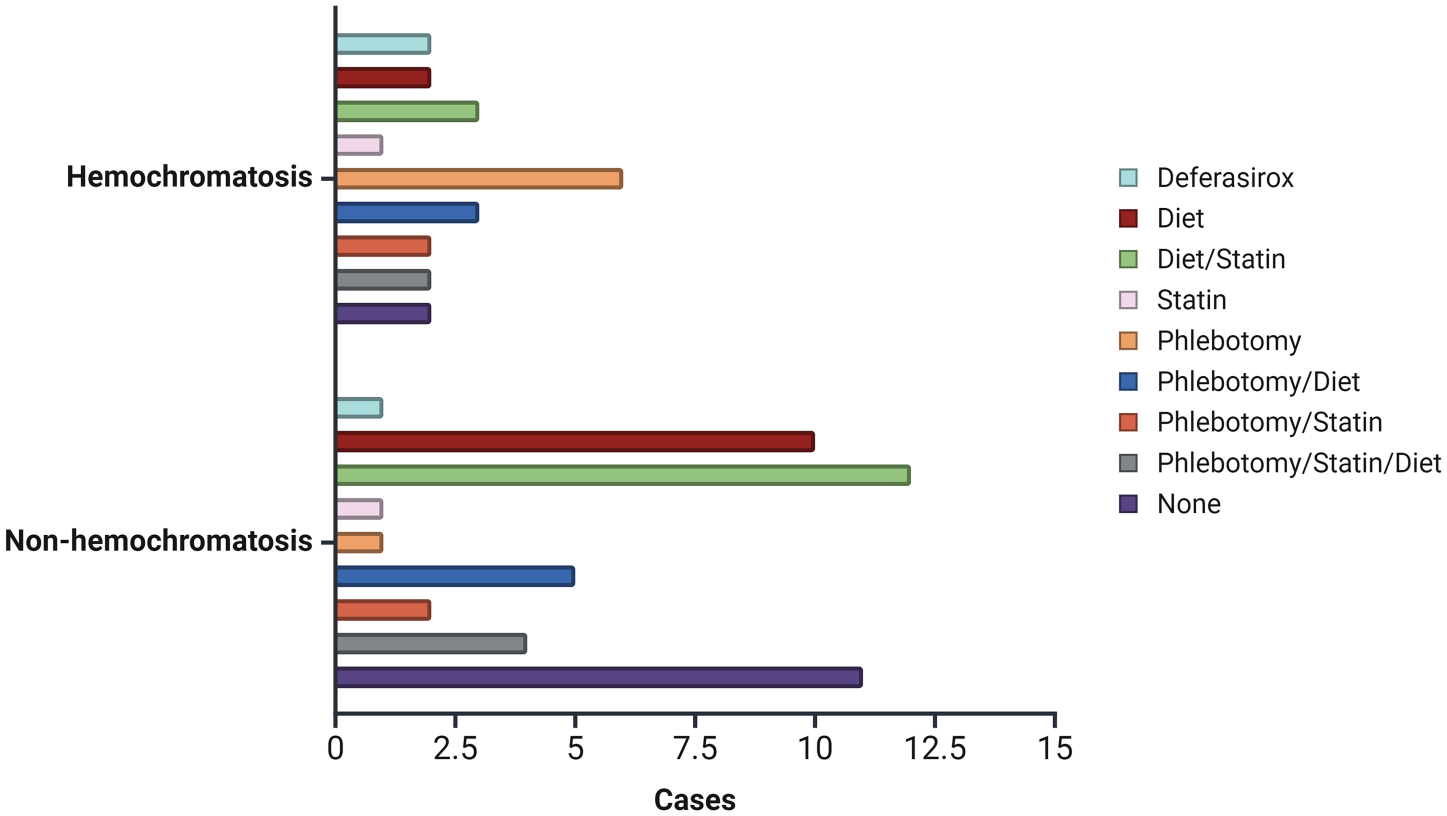
Distribution of treatment received between patients with and without a diagnosis of hemochromatosis.

Similar distributions were identified in terms of sex, history, and clinical symptoms between patients with and without hemochromatosis (*p* > 0.05; Table 3). No differences were identified concerning patient age; however, there was evidence of a longer follow-up in patients with hemochromatosis (5.9 ± 2.8 years) (*p* = 0.002; Table 4). Although not statistically significant association was found, a higher proportion of mild, moderate, and severe steatosis was found among patients without hemochromatosis (*p* > 0.05). Conversely, a predominance of both slight (100.0%) and limitrophe (58.3%) iron overload was identified among patients with hemochromatosis (*p* = 0.036; Table 3). In terms of treatment, Deferasirox (66.7%, *n* = 2) and phlebotomy plus dietary control (85.7%, *n* = 6) were predominant among patients with hemochromatosis (*p* = 0.048; Figure 1).

**Table 3:** Clinical and demographic variables associated with the diagnosis of hemochromatosis.

	Hemochromatosis diagnosis						
	Total	No		Yes		*p*- value*	OR (CI95%)
	*n*	*n*	%	*n*	%		
Sex							
Female *Ref.*	25	18	72.0	7	28.0	0.519	1,418 (0.489 to 4.116)
Male	45	29	64.4	16	35.6		
Diabetes Mellitus 2							
No *Ref.*	69	46	66.7	23	33.3	0.481	0.659 (0.025–16.822)
Yes	1	1	100.0	0	0.0		
Liver cirrhosis							
No *Ref.*	69	46	66.7	23	33.3	0.481	0.659 (0.025–16.822)
Yes	1	1	100.0	0	0.0		
Fatigue							
No *Ref.*	51	34	66.7	17	33.3	0.889	0.923 (0.298–2.854)
Yes	19	13	68.4	6	31.6		
Joint pain							
No *Ref.*	58	40	69.0	18	31.0	0.475	1.587 (0.443–5.682)
Yes	12	7	58.3	5	41.7		
Hepatic ultrasound							
Normal *Ref.*	10	6	60.0	4	40.0	0.554	
Mild hepatic stheatosis	24	16	66.7	8	33.3		0.750 (0.163–3.441)
Moderate hepatic stheatosis	9	8	88.9	1	11.1		0.187 (0.016–2.137)
Severe hepatic stheatosis	1	1	100.0	0	0.0		0.481 (0.015–14.702)
N/A	26	16	61.5	10	38.5		
Liver iron content							
Normal *Ref.*	18	11	61.1	7	38.9	0.036	
Slight overload	1	0	0.0	1	100.0		4.600 (0.164–128.547)
Limitrophe overload	12	5	41.7	7	58.3		2.200 (0.496–9.745)
N/A	39	31	79.5	8	20.5		

*p values were calculated from the Chi-square test.

**Table 4:** Clinical and analytical parameters associated with the diagnosis of hemochromatosis.

	Hemochromatosis diagnosis					Regression	
	No		Yes		*p*- value*	*b*	*p* value
	Mean	±SD	Mean	±SD			
Age at diagnosis (years)	56.0	13.5	56.4	11.7	0.901	−0.160	0.271
Current age (years)	59.5	13.3	62.3	11	0.360	0.157	0.285
Follow-up time (years)	3.5	3.0	5.9	2.8	0.002	0.004	0.258
Initial ferritin (μg/L)	746.7	626.5	1190.6	1682.6	0.242	0.004	0.056
Last Ferritin (μg/L)	634.8	428.5	329.0	248.5	0.001	−0.006	0.015
Initial Transferrin Saturation (mg/L)	42.5	24.4	137.5	378.6	0.317	0.004	0.906
Last Transferrin Saturation (mg/L)	37.2	15.0	43.7	11.6	0.132	0.018	0.155

*p values were calculated from student’s *t*-test.

Additionally, none of the demographic, clinical, or imaging findings were significantly associated with the diagnosis of hemochromatosis (Tables 3, 4). Among the serum indicators, the mean values of initial ferritin, initial transferrin saturation, and last transferrin saturation were higher among patients with hemochromatosis; however, these differences were not statistically significant. A lower mean final serum ferritin was identified among patients with hemochromatosis (329.0 ± 248.5 μg/L) (*p* = 0.001). Furthermore, the final ferritin measurement had a negative association with the diagnosis of hemochromatosis (*b* = −0.006, *p* = 0.015; Table 4).

**Figure 2:**
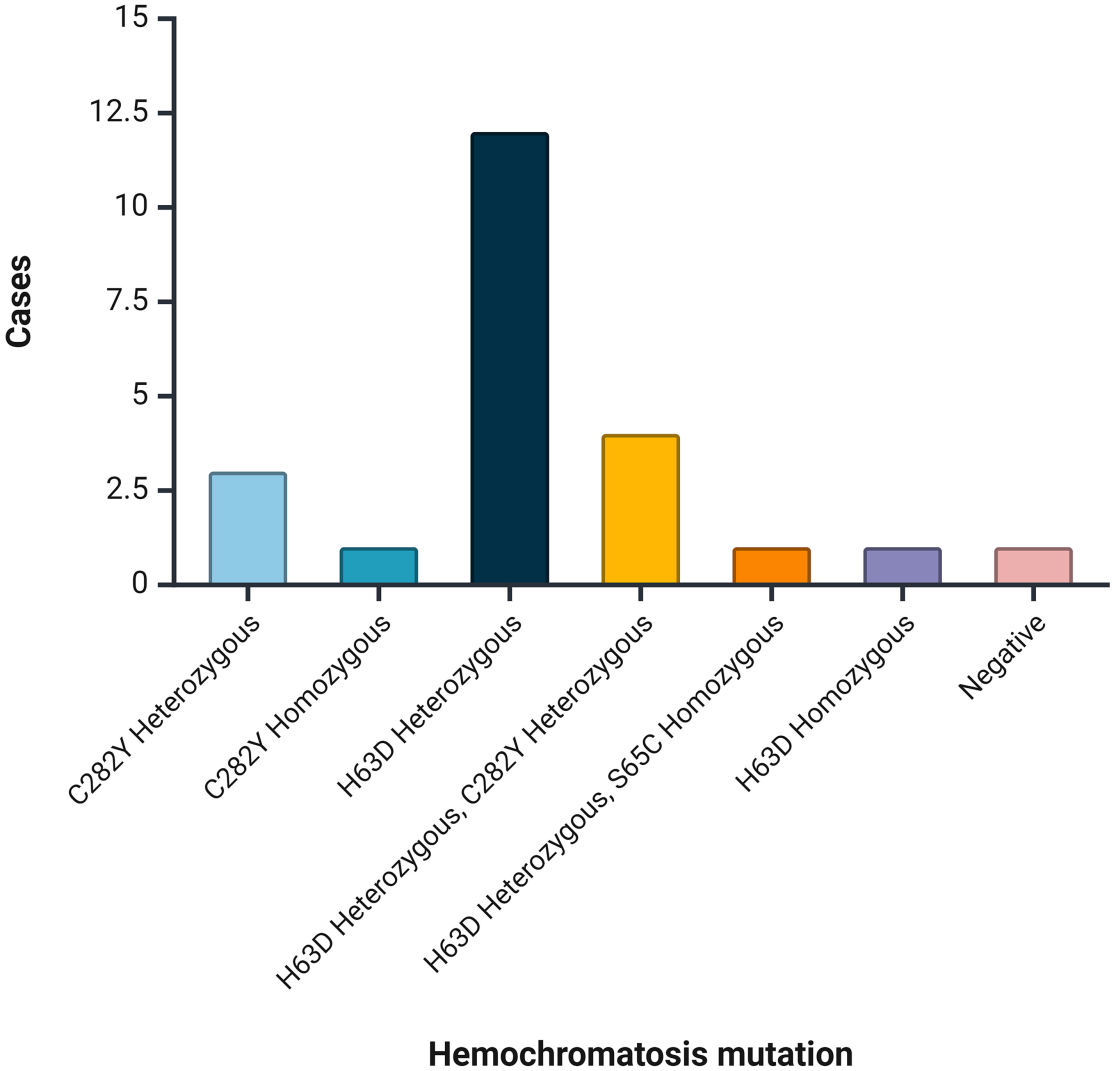
Mutations identified among patients with hemochromatosis.

Among patients with hemochromatosis (*n* = 23), mutations were found in 95.7% (*n* = 22). The most frequent mutation was H63D Heterozygous (52.2%, *n* = 12), followed by H63D Heterozygous + C282Y Heterozygous (17.4%, *n* = 4; Figure 2).

## 4 Discussion

This study presents a 10-year analysis of data from 70 patients diagnosed with iron overload who underwent genetic characterization. Male patients accounted for 64.1% of the cohort, representing a 1.8:1 male-to-female ratio, similar to the distribution reported in previous studies (3). This gender disparity may be influenced by menstruation in women, which serves as a natural source of iron loss, potentially delaying the onset of clinical symptoms and leading to later diagnoses in women of reproductive age (14).

The mean age at diagnosis in our study was 56.4 years (±12.8) in patients with hemochromatosis, compared to 56.0 years (±13.5) in the remaining patients with iron overload. Although penetrance varies by population, it has been shown to increase with age. Our findings suggest that the age of presentation in our participants is somewhat older than the commonly described range of the fourth and fifth decades of life (15, 16). This discrepancy might be attributed to challenges in accessing healthcare in developing countries like Colombia, leading to delays in diagnosis.

Fatigue was the most frequently reported symptom among all patients with iron overload, while joint pain predominated in those with hemochromatosis, consistent with existing literature (17, 18). Notably, the remaining patients in our sample were asymptomatic and were referred to hematology due to incidental findings of elevated iron or ferritin levels, which also aligns with literature showing that up to 18% of men and 5% of women may have hepatic iron overload without clinical symptoms (19).

While cardiac manifestations are rare in type 1 hemochromatosis, type 2 hemochromatosis—typically affecting younger patients (<30 years)—is more often associated with significant cardiac and endocrine complications. Cardiac iron overload in type 2 hemochromatosis can lead to both restrictive and dilated cardiomyopathy and is also linked to arrhythmias such as atrial fibrillation and sick sinus syndrome. These complications are thought to result from endothelial dysfunction, which increases carotid intima-media thickness and oxidative stress (20, 21). In our cohort of 70 patients, only one was diagnosed before age 30. This individual, a carrier of the C282Y/H63D mutation, remained asymptomatic for cardiovascular symptoms at the time of follow-up.

Diagnosis was based on elevated ferritin and transferrin saturation levels, with the mean initial transferrin saturation in our patients being approximately 70%. It is notable that transferrin saturation values of 45% or more can identify 97.9–100% of C282Y homozygotes (22). Screening is recommended solely for first-degree relatives of patients with type 1 hereditary hemochromatosis, as disease penetrance is influenced by both genetic mutation and shared environmental factors (20).

In HH, biochemical abnormalities, such as elevated transferrin saturation and ferritin levels, typically precede clinical symptoms, which can range from mild fatigue and arthralgia to severe conditions like hepatocellular carcinoma, diabetes mellitus, and cardiac abnormalities (23, 24). In our study, 41.7% of patients reported joint pain, and 31.6% experienced fatigue, which may support the notion of delayed diagnosis.

Hepatic steatosis was observed in nearly half of the patients, although its distribution across all grades (mild, moderate, and severe) was higher among those without a diagnosis of hemochromatosis, showing no significant association with HH. Although iron overload is known to affect lipid and glucose metabolism, contributing to insulin resistance and hepatic fat deposition, studies such as those by Hernaez et al. found no significant association between common HH genetic variants and metabolism dysfunction-associated fatty liver disease (MAFLD) (25).

Measuring liver iron content is essential for managing HH. While several invasive methods exist, MRI, particularly T2-weighted imaging, offers a noninvasive alternative for estimating iron deposition in the liver and spleen, aiding in differentiating between hereditary hemochromatosis and secondary iron overload (20, 26). In our sample, MRI demonstrated a predominance of mild to borderline hepatic iron overload among HH patients, though this finding was not statistically significant. A meta-analysis involving 819 HH patients reported a negative predictive value of 0.83 for MRI spin echo T2, indicating that while MRI is useful for ruling out HH, it has limited diagnostic power (27).

HH can be categorized based on the affected iron metabolism protein, with Type 1 being the most prevalent form. The most common mutation involves a G-to-A transition at nucleotide 845 of the HFE gene, leading to a cysteine-to-tyrosine substitution at amino acid position 282 (C282Y) (28). This mutation is most frequent in Caucasians of Northern European descent, where its prevalence can reach up to 6% and correlates with 80% of HH cases, and nearly 100% in the white non-Hispanic population from Australia (29–31). In contrast, information from South America reveals a prevalence between 3 and 14.3% for homozygous C282Y and close to 9% for heterozygous C282Y/H63D in Argentina (32, 33), and 21.6% for homozygous C282Y and 11.7% for heterozygous C282Y/H63D in Brazil (34). Of our 70 patients, 23 tested positive for the C282Y mutation based on hemochromatosis panel analysis of peripheral blood. However, this mutation is uncommon in Colombia, reflected in our study by only one homozygous patient and three heterozygous individuals for C282Y/H63D. The H63D mutation alone typically does not cause significant iron overload but may contribute to compound heterozygosity with C282Y (20). In our study, H63D heterozygotes and compound heterozygotes were the most common genotypes, often presenting with elevated iron indices, including transferrin saturation and serum ferritin. However, clinically significant iron overload remains rare among these patients. The S65C mutation, considered clinically insignificant, was the least common in our sample, consistent with literature that rarely associates it with a significant clinical phenotype, and was not found in the Argentinian population (20, 32). Currently, there are no established biological or clinical criteria defining what level of iron overload warrants further diagnostic testing, leaving the role of compound heterozygosity in HH diagnosis uncertain (21).

In our clinical setting, most patients with iron overload do not have an associated genetic mutation. Therefore, it is essential to consider secondary causes, such as hepatotropic viral infections, alcohol consumption, or MAFLD. For these cases, lifestyle modifications and interventions to reduce iron deposition are critical to prevent progression to cirrhosis. The findings of this study expand the limited literature on iron overload disorders and hemochromatosis in South America, particularly in Colombia, where detailed demographic and genetic data are scarce. These results provide a baseline for future research and highlight the need for improved access to timely healthcare to prevent complications arising from delayed diagnosis.

### 4.1 Limitations

This study has several limitations that should be acknowledged. As a retrospective observational analysis based on clinical records from a single specialty care center, the research is subject to inherent biases, including selection bias due to the nonprobability sampling method and the exclusion of records with insufficient data. The relatively small sample size may limit the statistical power to detect significant associations, and the findings may not be generalizable beyond the specific population served by Hemato-Oncologos S.A. in southwestern Colombia. Furthermore, the study’s focus on a limited number of genetic mutations means that other relevant genetic factors were not explored, potentially limiting the depth of genetic knowledge. These factors, combined with the geographic focus, suggest that the findings may not fully represent the broader population of patients with iron overload or hemochromatosis in Colombia, as well as in other nations in the region.

## 5 Conclusion

This study reveals substantial genetic diversity among Colombian patients with iron overload, marked by a low prevalence of the C282Y mutation and a higher prevalence of the H63D mutation, distinguishing their genetic profile from hereditary hemochromatosis patterns observed in European populations. Although these patients presented with elevated ferritin and transferrin saturation levels, no significant differences in clinical symptoms were observed, suggesting possible delays in diagnosis and an underestimation of early-stage cases. These findings highlight the importance of an early diagnostic approach tailored to regional genetic patterns to prevent long-term complications, such as cirrhosis. Future research should explore additional genetic mutations and further delineate the characteristics of hereditary hemochromatosis across diverse Latin American populations.

## Data Availability

The original contributions presented in the study are included in the article/supplementary material, further inquiries can be directed to the corresponding author/s.
